# Supplementary figures and images for: Immunoglobulin M regulates airway hyperresponsiveness independent of T helper 2 allergic inflammation
Source: eLife. 2025 Dec 16;12:RP90531. doi: 10.7554/eLife.90531 (PMC12707823; doi:10.7554/eLife.90531)

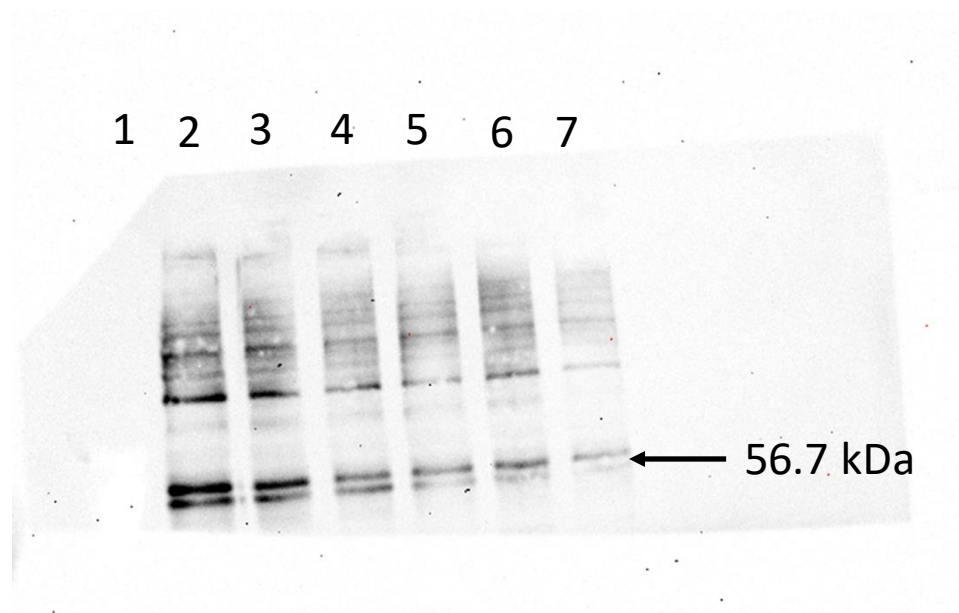

- 1 – PageRuler™ Plus Prestained protein ladder (10-250 kDa)
- 2 – WT HDM 1
- 3 – WT HDM 2
- 4 – WT HDM 3
- 5 – IgM KO HDM 1
- 6 – IgM KO HDM 2
- 7 – IgM KO HDM 3

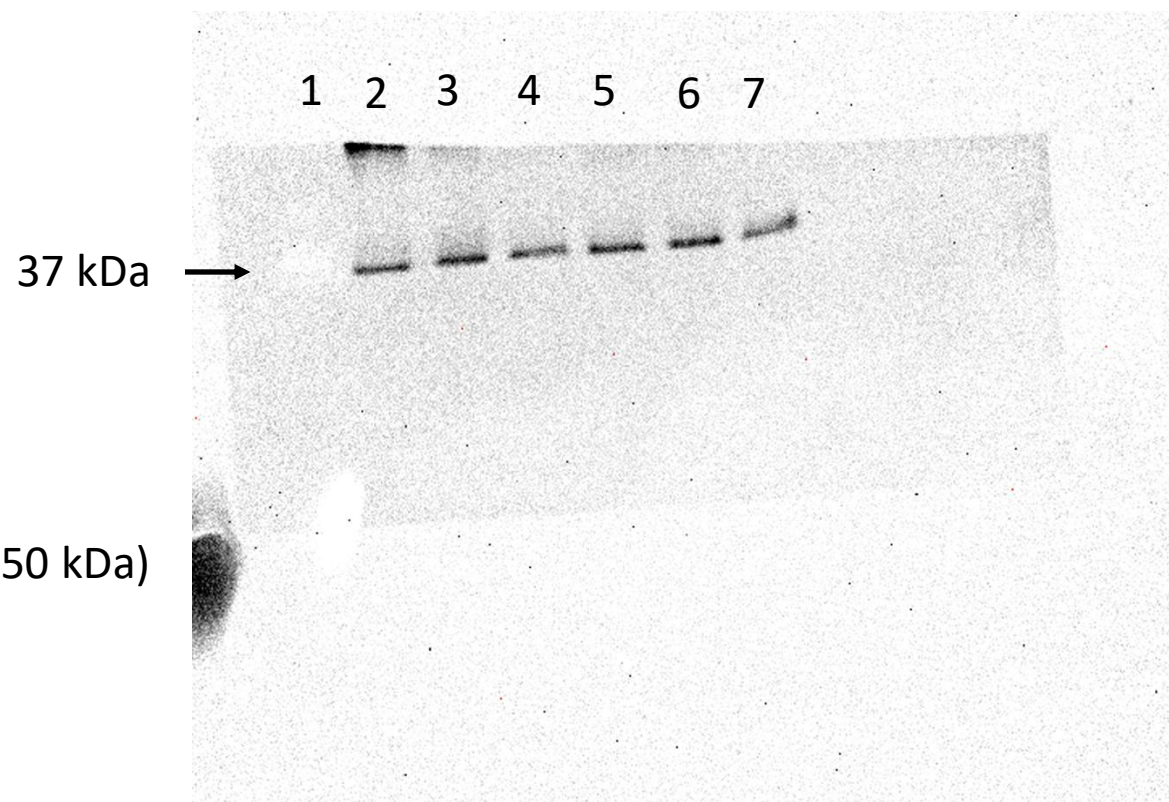

Supplement: Figure 5—source data 1. [file elife-90531-fig5-data1.zip › Figure_5_-source_data_1.pdf]

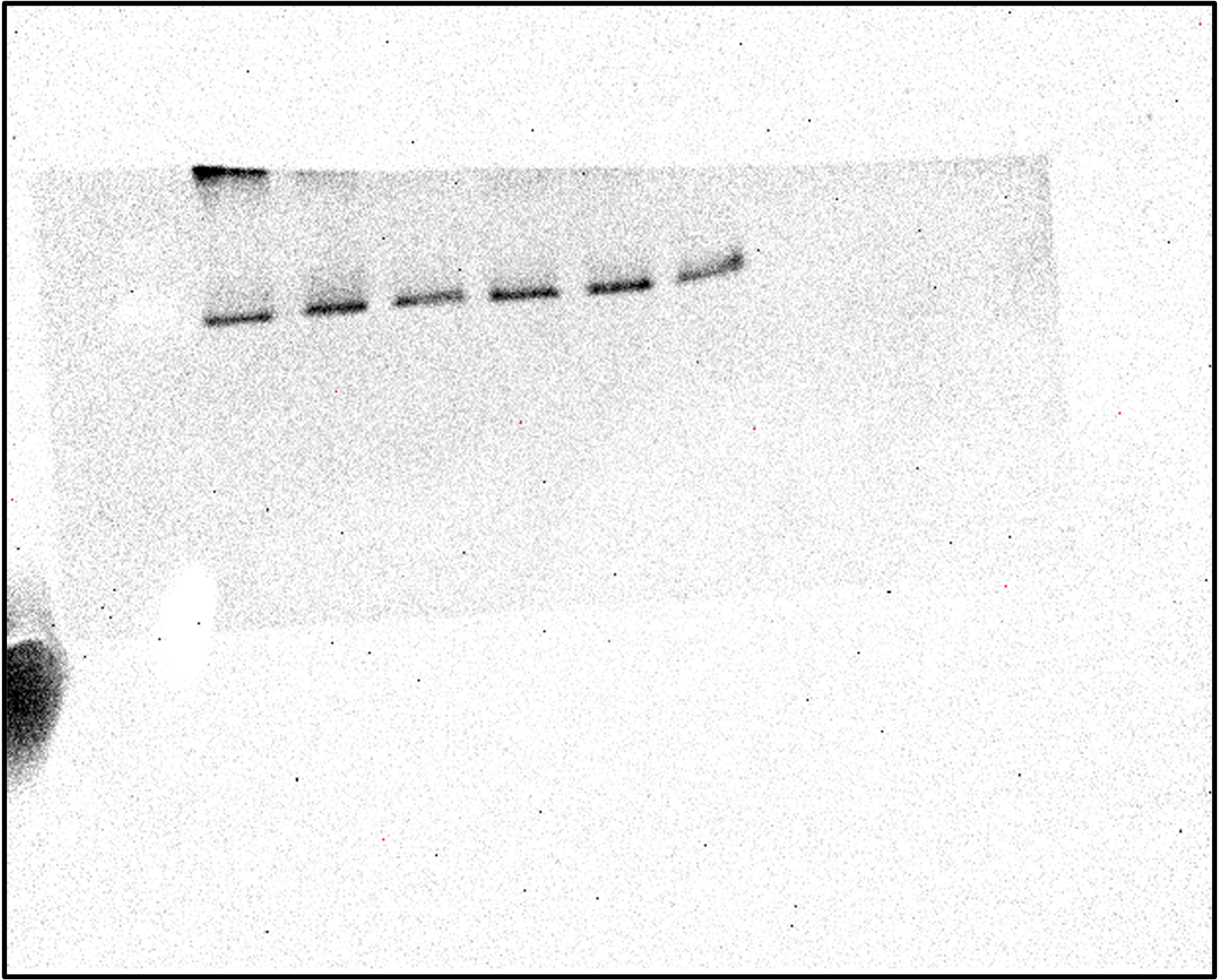

Supplement: Figure 5—source data 2. [file elife-90531-fig5-data2.zip › GAPDH.png]

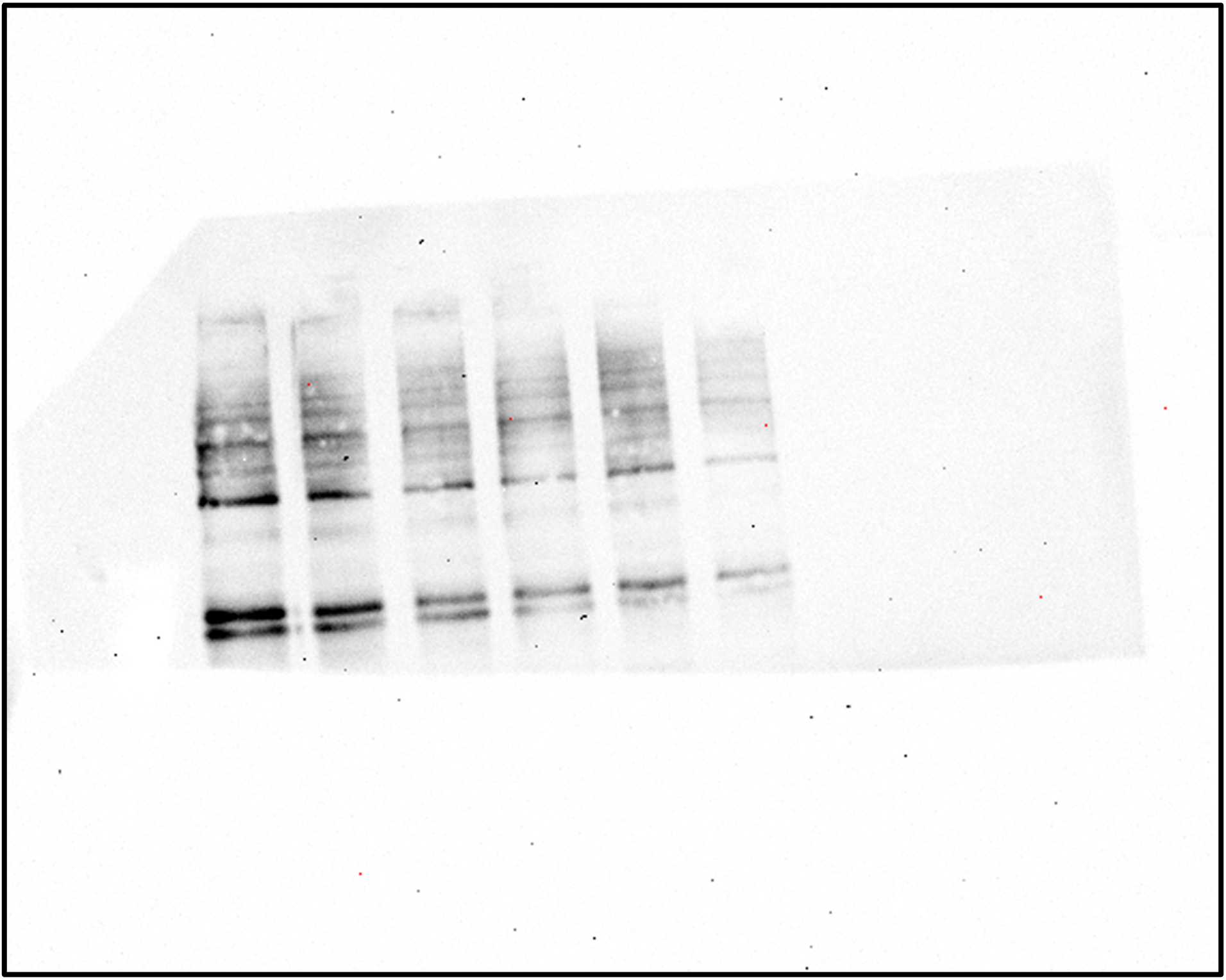

Supplement: Figure 5—source data 2. [file elife-90531-fig5-data2.zip › BAIAP2L1.png]

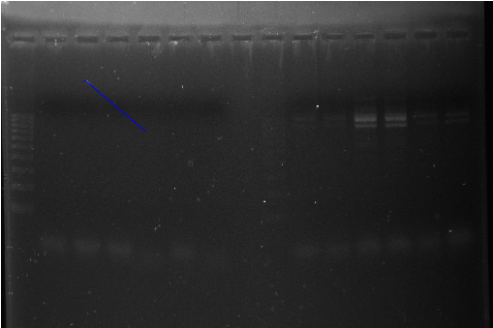

Supplement: Figure 6—figure supplement 1—source data 2. [file elife-90531-fig6-figsupp1-data2.zip › DNA_gel_sg_RNA_BAIAP2L1.png]
