# Supplementary material for: Immunoglobulin M regulates airway hyperresponsiveness independent of T helper 2 allergic inflammation
Source: eLife. 2025 Dec 16;12:RP90531. doi: 10.7554/eLife.90531 (PMC12707823; doi:10.7554/eLife.90531)
Supplement: Figure 6—figure supplement 1—source data 1. [file elife-90531-fig6-figsupp1-data1.zip › Figure_6_-figure_supplement_1_source_data_1.pdf]

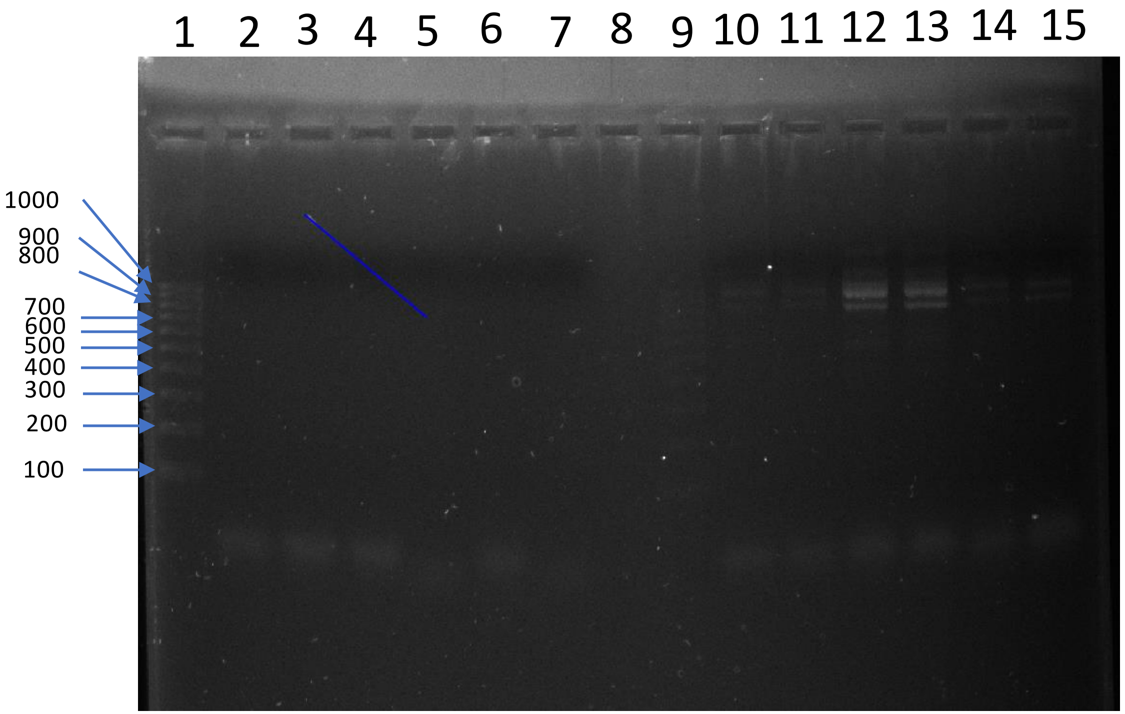

Primer set 1

1 – DNA ladder 1000bp
2 – unstimulated Scramble
3 – Unstimulated BAIAP2L1
4 – TNF-α Scramble
5 – ACh Scramble
6 – TNF-α BAIAP2L1
7 – ACh BAIAP2L1
8 – skipped

Primer set 2

9 – DNA ladder 1000bp
10 – unstimulated Scramble
11 – Unstimulated BAIAP2L1
12 - TNF-α Scramble
13 – ACh Scramble
14 – TNF-α BAIAP2L1
15 – ACh BAIAP2L1
